# Mayer-Rokitansky-Küster-Hauser syndrome type II. Case report

**DOI:** 10.5935/1518-0557.20250057

**Published:** 2025

**Authors:** Juan José Reinoso Calle

**Affiliations:** 1 Department of Medicine, Universidad Católica de Cuenca, Azuay, Ecuador

**Keywords:** paramesonephric ducts, scoliosis, fused kidney,46, XX disorders of sexual development

## Abstract

Mayer-Rokitansky-Küster-Hauser syndrome is a rare congenital disorder characterized by Müllerian agenesis while preserving secondary sexual characteristics and a normal 46XX karyotype. It is classified into two types, with type II involving additional multisystemic anomalies affecting the skeletal, renal, and cardiovascular systems. Due to its rarity, epidemiological data on prevalence and incidence are lacking, and no cases have been previously reported in our country. We present the case of a 48-year-old female patient diagnosed with Mayer-Rokitansky-Küster-Hauser syndrome type II at the age of 17. Her medical history includes atrial septal defect, agenesis of the uterus and two-thirds of the vaginal canal, amorphous ovarian tissue, renal ectopia and fusion in the pelvis, supernumerary rib, dorsal scoliosis, fused vertebrae, butterfly vertebra, and annular pancreas. Despite these internal anomalies, her external genitalia are normal, and secondary sexual characteristics are preserved. In 2002, she underwent a McIndoe vaginoplasty to create a functional vaginal canal. This case highlights the complex etiology of MRKH type II syndrome, involving genetic and epigenetic factors, particularly HOX and WNT genes. Diagnosis was confirmed through computed tomography, revealing characteristic anomalies. Treatment included surgical intervention with vaginoplasty and progressive dilation, leading to satisfactory anatomical function and improved body perception. Additionally, cognitive-behavioral therapy played a crucial role in reinforcing the patient’s identity and self-esteem.

## INTRODUCTION

Mayer-Rokitansky-Küster-Hauser syndrome (MRKH) is a rare disorder in the medical literature (Sfar *et al.*, 2024). It is a congenital alteration in the development of the paramesonephric ducts (Müllerian ducts) in their stage of sexual undifferentiation, which occurs in the sixth week of embryonic development (Briosa *et al.*, 2019). This alteration affects the formation of the reproductive tract and results in a 46XX female sexual phenotype, leading to agenesis or aplasia of the internal genitalia (Liszewska-Kapłon *et al.*, 2020).

MRKH is divided into two types: Type I, which is characterized by uterine and vaginal aplasia in the upper two-thirds, with a prevalence of 1/4,500 live-born girls, and Type II, which also includes extragenital changes affecting organs at the renal, cardiovascular and skeletal levels (Briosa *et al.*, 2019). Clinical manifestations may include scoliosis and Klippel-Feil syndrome (Kisu *et al.*, 2019). The prevalence of this pattern of multiple anomalies is unclear. Estimates range from 1 in 4,500 live-born girls to 1 in 4,500,000 live-born girls (Bleach & Vogt, 2020; Triantafyllidi *et al.*, 2022).

The etiology of the syndrome is idiopathic and the main theory focuses on genetics with an autosomal dominant inheritance (Kisu *et al.*, 2019). Differences in development between twins suggest that epigenetic alterations may be responsible, with a prevalence of 1-5% in relatives in the first instance (Pai & Shakir, 2013). In addition, estrogen overexposure and abnormal HOXA expression along with activation of the anti-Müllerian hormone (AMH) promoter are considered to be contributing factors (Zeng *et al.*, 2022).

At the continental level, three cases have been reported in the United States over the last five years (Bleach & Vogt, 2020). In Europe, three cases have been reported in the UK and two in Spain, according to recent studies (Triantafyllidi *et al.*, 2022). However, a review of the medical literature has not identified specific figures for Latin America.

The diagnosis of MRKH is based on imaging findings, which are typified as type I or II using computed tomography and magnetic resonance imaging, the latter being the gold standard (Romero *et al.*, 2022). Treatment for MRKH syndrome type II may vary depending on the specific needs of each patient. Vaginal dilatation is the initial treatment of choice to create a functional neovagina using non-surgical techniques. However, when dilatation is not effective or feasible, surgical interventions such as vaginoplasty are considered, or alternatively, a mixed technique could be chosen (Chmel *et al.*, 2021; Pfeifer, 2021; Yang *et al.*, 2021).

The present study focuses on the treatment of patients with Mayer-Rokitansky-Küster-Hauser syndrome (MRKH) type II, with special attention to the creation of a functional vaginal canal and the psychosocial implications of the condition. The therapeutic approach aims to offer patients as normal a life as possible, tailoring treatment according to the specific MRKH typing (Herlin, 2024). Treatment options include minimally invasive interventions and surgical procedures, selected after a personalized assessment that considers the anatomical malformations present (Kyei-Barffour *et al.*, 2021).

It is imperative to assess the psychological and sexual impact associated with the anatomical variations of the syndrome, as women with MRKH face challenges that affect their female identity, which can lead to disorders such as depression, anxiety, and decreased self-esteem (Both *et al.*, 2018; Odhaib *et al.*, 2021). Therefore, treatment should not be limited to physical aspects, but should also encompass psychosocial support to ensure a holistic approach, promoting both the physical and emotional health of patients. A comprehensive approach is essential for ensuring long-term well-being and adaptation (The American Fertility Society, 1988).

This research work is of particular importance due to the exceptional rarity of MRKH syndrome type II, a pathology with very low prevalence and few reports worldwide. This case is unique in that it presents a complete phenotype, allowing for in-depth exploration of its clinical manifestations and multisystemic involvement, including the genitourinary, renal, cardiovascular, and skeletal systems. Disseminating this report will provide a valuable resource for managing future patients with this condition.

## CASE DESCRIPTION

Female patient, 48 years old, of mixed race, teacher, diagnosed with Mayer-Rokitansky-Küster-Hauser syndrome type II at 17 years of age, under study for primary amenorrhea. She has a family medical history of epileptic mother and cousin with infertility. A history of ischemic CVD and bilateral thalamic infarction was assessed.

**Figure 1:**
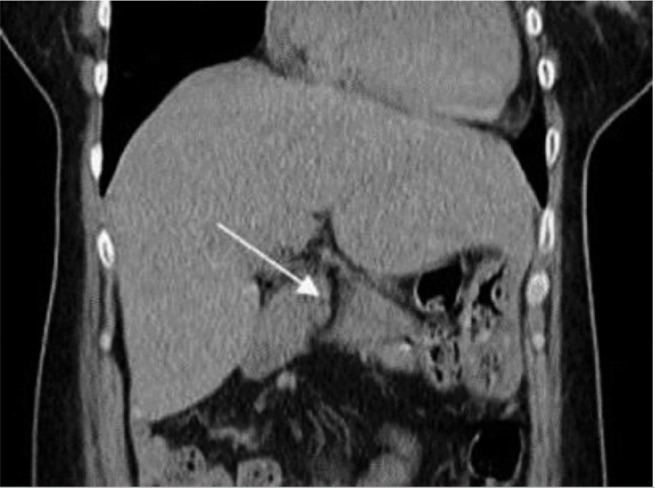
Pancreatic assessment. Coronal CT scan reveals the presence of a hypodense tubular structure that crosses the pancreas longitudinally, with loss of usual morphology with pancreatic tissue on both sides of the duodenum compatible with annular pancreas.

**Figure 2:**
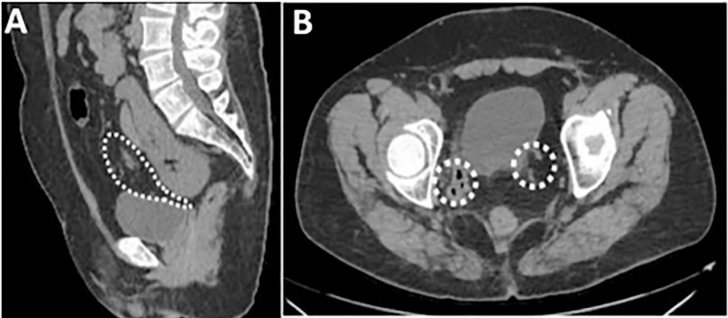
Internal sexual organs: (A) CT sagittal section, showing the absence of uterine (B) CT axial section, 28*27mm axial section, which reinforces that it is located in the contour of the cecum, compatible with ovarian tissue.

In the clinical findings, an atrial septal defect (ASD) of the ostium secundum type was identified, resulting in congestive hepatomegaly (Figure 1). In the evaluation of the internal sexual organs, the computed tomography (CT) in sagittal section shows the complete absence of the uterus and the upper two thirds of the vaginal canal (Figure 2A) and in axial sections an amorphous tissue of 28x27 mm with reinforcement is identified, located in the contour of the cecum, indicative of ovarian tissue (Figure 2B).

**Figure 3:**
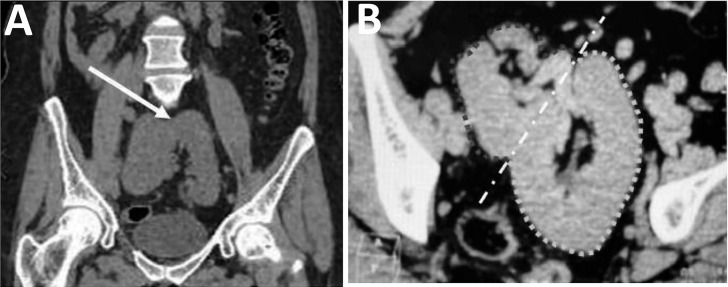
Renal assessment: (A) Coronal CT scan showing a renal ectopia together with an abnormal rotation of the kidney, with apparent fusion of the kidney in the pelvis, (B) Axial CT scan clearly showing the renal ectopia, with the lobulated kidney occupying an abnormal position in the pelvis. The fusion of the kidney is confirmed in this section, where the anteriorly directed hilum is observed.

**Figure 4:**
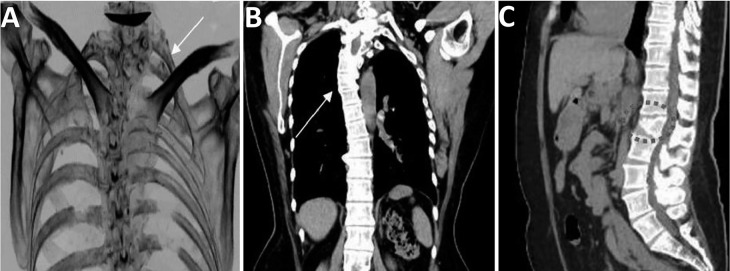
Bone assessment. (A) CT bone window, left supernumerary rib in the seventh cervical vertebra (B) CT coronal slice, high dorsal scoliosis with right concavity vertebral fusion in the c5/c6 vertebrae (C) CT sagittal slice, vertebra with butterfly appearance in the lumbar region (L2).

In the renal evaluation, CT in coronal and axial sections shows a renal ectopia with abnormal rotation of the kidney, highlighting its fusion in the pelvis with the hilum directed anteriorly (Figure 3A-B). In the bone analysis, the bone window CT reveals the presence of a left supernumerary rib in the seventh cervical vertebra (Figure 4A), while the coronal sections show high dorsal scoliosis with concavity to the right and vertebral fusion in the C5/C6 vertebrae (Figure 4B) and a vertebra with a butterfly appearance in the lumbar region (L2) (Figure 4C).

**Figure 5:**
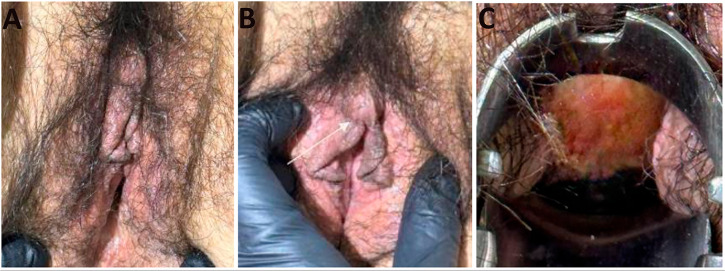
Genital assessment (A-B) An evaluation of the external sexual organs is performed. Hypertrophy of the labia minora (C) The internal part of the vaginal canal is observed, absence of cervix, the fundus of the vaginal canal is evaluated as a smooth structure, revealing a vaginal canal of 11 cm in length, result of the McIndoe technique.

**Figure 6:**
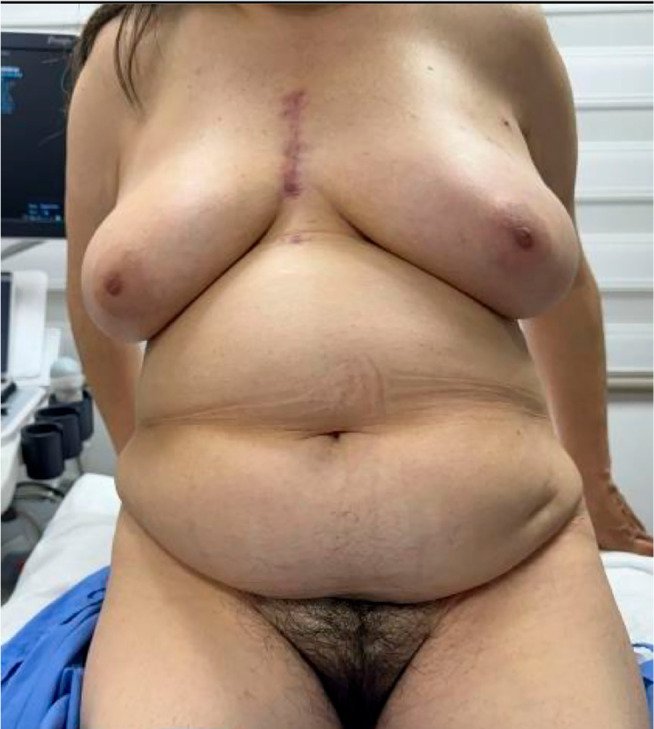
Presence of secondary sexual characteristics, including breast development and normal pubic hair distribution, is observed.

Finally, in the assessment of the pancreas, the coronal CT scan highlights a hypodense tubular structure longitudinally crossing the pancreas (Figure 1), indicating the presence of an annular pancreas with loss of usual morphology. The external genitalia exhibit a normal anatomy (Figure 5A-B), however, as a sequel of MRKH syndrome type II, the patient underwent in 2002, at 26 years of age, a vaginoplasty with the aim of building a functional vaginal canal for copulation, in (Figure 5C) the bottom of the vaginal canal is visualized, where a vaginal canal of 11 cm in length is appreciated, obtained by applying the McIndoe technique. Additionally, the presence of secondary sexual characteristics, including breast development and normal pubic hair distribution, is observed (Figure 6).

She presented a depressive picture as a result of the psychological and emotional implications of her disease. The congenital absence of internal female reproductive structures contributed to a profound sexual identity crisis, affecting her self-perception and self-esteem. These factors, added to the social stigma and the lack of adequate psychological support, triggered episodes of suicidal ideation and self-harm attempts, evidencing the need for a comprehensive approach that includes specialized psychological support.

The surgical intervention not only allowed him greater anatomical functionality, but also significantly improved his body perception, helping to alleviate dysphoria related to his sexual identity. In parallel, cognitive behavioral therapy (CBT) addressed issues of self-esteem, depression and anxiety.

The results of the case report provide a comprehensive overview of the anatomical complexities linked to MRKH syndrome type II, underscoring the relevance of a multidisciplinary evaluation to address the various clinical manifestations of this condition.

## DISCUSSION

During early embryonic development, two pairs of genital ducts are present: the mesonephric ducts of Wolff and the paramesonephric ducts of Müller, in embryos with a female karyotype, the absence of antimullerian hormone (AMH) and testosterone, normally regulated by the SRY gene in the male sex, allows the development of the Müllerian ducts (Chmel *et al.*, 2021; Yang *et al.*, 2021). These ducts then fuse to form the uterus, fallopian tubes, and the upper part of the vagina. However, in Mayer-Rokitansky-Küster-Hauser syndrome (MRKH), this process is disrupted, resulting in agenesis or hypoplasia of the uterus and the upper two-thirds of the vagina. While the ovaries and fallopian tubes typically develop normally, enabling ovarian function and the development of secondary sexual characteristics, extragenital anomalies, such as renal and skeletal malformations, are characteristic of MRKH type 2 (Briosa *et al.*, 2019; Qiu *et al.*, 2021).

The potential causes and associated etiopathogenic factors that explain the development of the syndrome are subject to diverse perspectives. One of the most widely recognized perspectives is alterations in the HOX gene family. These genes encode a set of transcription factors distinguished by their homeodomain, a key structure for their function in the regulation of morphogenesis, cell differentiation, and the identity of body segments along the anterior-posterior axis in embryonic development (Thomson *et al.*, 2023). Studies have identified mutations in the HOXA9 and HOXA10 genes as a contributing factor to abnormalities in uterine and fallopian tube differentiation, while HOXA7 has been linked to uterine formation and HOXD9-13 has been associated with skeletal and renal alterations (Dube *et al.*, 2023; Deshmukh *et al.*, 2024).

Recent studies have emphasized the significance of the WNT gene family, which encodes signaling proteins in vertebrates. Specifically, alterations in WNT4 have been linked to uterine and vaginal agenesis, and WNT9B to renal abnormalities (Thomson *et al.*, 2023). A study of Chinese women with Müllerian duct malformations identified mutations in WNT9B, results that were replicated in a Caucasian cohort (Dube *et al.*, 2023; Gaikaiwari *et al.*, 2024). In addition, studies in rodents have proposed a digenic model involving WNT9B, LHX1, and TBX6 (Kyei-Barffour *et al.*, 2021). Other genes, such as WT1, have been implicated in renal and genital malformations, while PAX2 affects renal structuring, and PBX1 is associated with congenital anomalies of the kidney and urinary tract syndrome, which can occur with or without hearing loss (Drusany Starič *et al.*, 2023; Ozkan *et al.*, 2023).

The observed divergence in monozygotic twins, where only one of them has MRKH syndrome, supports the theory that epigenetic factors could play a crucial role in the development of this condition (Kyei-Barffour *et al.*, 2021). In contrast to clearly identified genetic mutations, such as those affecting the HOX or WNT genes, epigenetic changes, such as overexposure to estrogens or abnormal expression of HOXA genes, as well as activation of the antimullerian hormone (AMH) promoter, suggest a more complex and less predictable mechanism that could contribute to the development of MRKH syndrome (Thomson *et al.*, 2023).

The present MRKH type II case report is of great relevance in the medical literature. It highlights an exceptionally complex clinical presentation, marked by multiple extragenital anomalies that transcend the typical manifestations of this condition. Unlike other documented cases of MRKH type 2, which present with extragenital alterations and uterine and vaginal agenesis, this particular case does not. The absence of these structures reveals not only their absence but also significant renal, skeletal, and cardiovascular malformations, which underscores the rarity of the case.

The diagnostic process commences with a physical evaluation encompassing an examination of the external genitalia and an internal inspection. To ensure a precise diagnosis, it is advisable to incorporate imaging studies, with magnetic resonance imaging (MRI) being the preferred method to detect uterovaginal agenesis in patients with Mayer-Rokitansky-Küster-Hauser (MRKH) syndrome. MRI provides a more detailed visualization of the müllerian structures compared to computed tomography (CT), which only provides general images (Kapczuk & Kędzia, 2021; Mahey *et al.*, 2023; Ozkan *et al.*, 2023). While MRI is the ‘Gold Standard’ for identifying uterine remnants or complete agenesis, the use of thoracoabdomino-pelvic CT remains necessary in cases where complementary studies are required to identify other structural malformations or agenesis associated with MRKH syndrome type 2, which may involve renal and skeletal abnormalities (Ekici *et al.*, 2013).

A thorough evaluation of the pelvic floor was conducted as part of the preliminary report, encompassing a meticulous examination of the external and internal sexual organs. This evaluation confirmed the absence of the vaginal canal. To complement this diagnostic assessment, a thoracoabdomino-pelvic computed tomography (CT) scan was performed, which substantiated the diagnosis of uterine agenesis. Additionally, the CT scan revealed the presence of various extragenital anomalies, including renal, cardiovascular, and skeletal abnormalities.

Although magnetic resonance imaging (MRI) is often considered the reference method for evaluating the pelvic structures and determining the presence of remnants of Müllerian ducts, it was not necessary to resort to this study in this case. The information obtained through CT was sufficient to establish a definitive diagnosis. Both uterine agenesis and associated anomalies were considered major diagnostic criteria, which allowed us to clearly confirm the presence of MRKH type 2 syndrome without the need for additional testing.

The clinical management of MRKH type 2 necessitates a multidisciplinary approach due to the numerous areas of involvement for the patient. In such cases, imaging studies play a pivotal role in planning treatment and follow-up, which may include reconstructive surgical interventions to enhance the quality of sexual and reproductive life for affected patients, contingent on their specific clinical features.

The primary objective is to empower patients by offering them the option to select a functional vaginal canal, this approach encompasses a range of interventions, including minimally invasive procedures and surgical treatments (Romano *et al.*, 2021). The selection of the most appropriate course of action is determined by a comprehensive evaluation of each case, taking into account the presence of malformations associated with MRKH (Singh *et al.*, 2022). The American College of Obstetricians and Gynecologists (ACOG) recommends the use of vaginal dilators as a first-line treatment due to its >93% success rate, surgical intervention to create a neovagina is recommended as a second-line treatment due to its greater complexity and results (Mahey *et al.*, 2023).

McIndoe’s method is the most common, with a high percentage of functional success, although it requires careful postoperative follow-up and carries the risk of complications such as graft errors or fistulas. Sigmoid vaginoplasty, on the other hand, despite avoiding dilatation, presents significant issues, including persistent discharge and the potential for adenocarcinomas (Singh *et al.*, 2022; Dube *et al.*, 2023; Gaikaiwari *et al.*, 2024). Laparoscopic procedures, such as the modified Vecchietti and Davydov, are distinguished by their reduced morbidity, reduced surgery times, and success rates close to 95%, making them preferred alternatives in many situations (Pinto *et al.*, 2022).

The patient was treated with a combination of the McIndoe technique and vaginal dilators, with the procedure performed in a progressive manner. Her postoperative evolution was favorable, with no complications. The patient indicated that she was able to lead a full and satisfactory sexual life after the intervention, without experiencing any difficulties or discomfort associated with the procedure.

With regard to the extragenital anomalies observed, characteristic of MRKH syndrome type 2, it was determined that surgical intervention was not necessary, since they did not affect the patient’s daily life or compromise her functionality. Alterations such as renal and skeletal malformations were monitored, but did not require additional treatment. The only exception was the correction of the atrial septal defect, which did require attention due to its potential impact on cardiovascular health.

An anatomical evaluation is necessary to diagnose MRKH type 2 syndrome, given its impact on a patient’s sexual and reproductive lives (Bleach & Vogt, 2020; Triantafyllidi *et al.*, 2022). This evaluation should include psychological and sexual aspects, as well as a comprehensive assessment of the patient’s identity and well-being (Gaikaiwari *et al.*, 2024).

During her puberty and after receiving a diagnosis of MRKH type 2, the patient began to experience profound gender dysphoria. She felt that the impossibility of having a uterus and menstruating, something she considered inherent to the female experience, distanced her from her gender identity. This emotional conflict manifested itself in feelings of inadequacy and anxiety, especially when comparing herself to other women around her. The absence of reproductive characteristics, such as the ability to bear children, contributed to her sense of not fully aligning with traditional female roles, generating a significant psychological impact on her well-being.

To address the patient’s dysphoria, a specialized psychological treatment plan was implemented. This treatment plan included body acceptance therapies and the reinforcement of her feminine identity, emphasizing non-biological aspects that contribute to femininity. Through cognitive-behavioral therapy, the patient focused on redefining her relationship with her body and her perception of what it means to be a woman. This treatment helped her rebuild a solid sense of self-esteem.

After a period of treatment, the patient demonstrated an ability to overcome emotional challenges and adopt a new perspective on her identity and life. She entered into a marital union with her partner, and together they initiated a family, providing care for her husband’s child. This process served to reinforce her sense of belonging and completeness, underscoring the notion that her value and capacity to love transcend any physical limitations.
